# Potassium and Obesity/Metabolic Syndrome: A Systematic Review and Meta-Analysis of the Epidemiological Evidence

**DOI:** 10.3390/nu8040183

**Published:** 2016-03-25

**Authors:** Xianlei Cai, Xueying Li, Wenjie Fan, Wanqi Yu, Shan Wang, Zhenhong Li, Ethel Marian Scott, Xiuyang Li

**Affiliations:** 1 Institute of Environmental Medicine, Zhejiang University, Hangzhou 310058, China; caixianlei@zju.edu.cn; 2 Ningbo Medical Treatment Center Lihuili Hospital, Ningbo 315000, China; 3 Department of Clinic Medicine, Zhejiang University, Hangzhou 310058, China; sherry98983@gmail.com; 4 Department of Epidemiology and Biostatistics, Zhejiang University, Hangzhou 310058, China; 3090103824@zju.edu.cn (W.F.); 3100102431@zju.edu.cn (W.Y.); wangsh2046@163.com (S.W.); 5 School of Civil Engineering and Geosciences, Newcastle University, Newcastle-upon-Tyne NE1 7RU, UK; Zhenhong.Li@newcastle.ac.uk; 6 School of Mathematics and Statistics, University of Glasgow, Glasgow G12 8QW, UK; Marian.Scott@glasgow.ac.uk

**Keywords:** potassium, obesity, metabolic syndrome, systematic review, meta-analysis

## Abstract

The objective of this study was to investigate the associations between potassium and obesity/metabolic syndrome. We identified eight relevant studies and applied meta-analysis, and nonlinear dose-response analysis to obtain the available evidence. The results of the pooled analysis and systematic review indicated that high potassium intake could not reduce the risk of obesity (pooled OR = 0.78; 95% CI: 0.61–1.01), while serum potassium and urinary sodium-to-potassium ratio was associated with obesity. Potassium intake was associated with metabolic syndrome (pooled OR = 0.75; 95% CI: 0.50–0.97). Nonlinear analysis also demonstrated a protective effect of adequate potassium intake on obesity and metabolic syndrome. Adequate intake of fruits and vegetables, which were the major sources of potassium, was highly recommended. However, additional pertinent studies are needed to examine the underlying mechanism.

## 1. Introduction

Central obesity has become a worldwide problem within the last several decades, and the prevalence of obesity remains high. More than 33% of adults and 17% of youths in the United States [1], and 26.3% of adults in Germany, where the prevalence is among the highest in Europe [2], are obese. Furthermore, the rest of the world is quickly catching up, particularly developing countries [3]. Metabolic syndrome (MetS), which is known as a cluster of obesity-driven alterations, such as central obesity, insulin-resistance, dyslipidaemia, and hypertension [4], has received increasing attention in recent years as its prevalence and public health burden have increased worldwide [5]. To date, substantial evidence shows that MetS is associated with diabetes [6,7], dyslipidaemia, cardiovascular disease [7,8], specific types of cancer [3,9], and many other conditions.

Potassium is anintracellular cationic electrolyte that is necessary for normal cellular function. Because it is easily excreted by the kidneys rather than stored in the body, humans need a constant supplement of potassium. However, the average potassium consumption is inadequate, only 54% and 58% compared to the recommended amount in the U.S. and Korean populations [10]. According to recent studies, the associations between potassium and obesity/MetS were investigated, although these observational studies are considered controversial. Several studies have shown that higher potassium could alleviate obesity and MetS risk, while other studies have found that potassium has a null effect on obesity and MetS.

To resolve the controversies and to improve the generalizability of these results, we conducted a systematic review and meta-analysis to combine information to estimate the overall effect of potassium on the risk of obesity and MetS.

## 2. Methods

### 2.1. Search Strategy

We searched for relevant articles that described the associations between potassium and obesity/MetS from medical and biological databases (Medline 1995–October 2015; Embase 1995–October 2015), using the following search terms (“potassium” or “potassium intake” or “urinary potassium”) and (“metabolic syndrome” or “MetS” or “obesity” or “obese”). We also scanned reference lists of relevant original studies and reviews to identify potential publication. Two investigators (Xianlei Cai and Xueying Li) performed the literature search independently.

### 2.2. Study Selection

Inclusion criteria were applied as the following: the study must (1) describe the association between potassium and obesity/MetS; (2) be an original study published in English prior to October 2015; and (3) have key data for meta-analysis or dose-response analysis. The following types of studies were excluded: (1) those not involved with exposure-response associations between potassium and obesity/MetS; (2) cytological studies, animal studies and reviews; and (3) low-quality articles.

### 2.3. Data Extraction

All data were independently extracted by two reviewers (Xianlei Cai and Xueying Li). The characteristics of the identified articles were extracted in the following manner: first author name, year of publication, study country, age, number of participants, number of cases, measurement of potassium, gender, outcome (obesity or MetS), odds ratio (OR) at the highest compared with the lowest category of potassium exposure and its 95% confidence interval (CI), and adjustment of confounding variables. The quality of the studies was assessed by two investigators (Xianlei Cai and Xiuyang Li) using the Newcastle-Ottawa scale [11]. The maximum score was nine stars. We regarded scores of 0–3 stars, 4–6 stars, and 7–9 stars as low, moderate and high quality, respectively. Through discussion, all discrepancies were resolved, and a final consensus was reached.

### 2.4. Statistical Analyses

The multivariate-adjusted ORs and 95% CI from original studies were extracted. If studies only provided 2 × 2 table data, then we calculated the ORs. Pooled ORs with 95% CI were calculated using a fixed or random effects model. When the result of the Q-test showed evidence for heterogeneity (*p* ≤ 0.05), we used a random effect model, as previously described by DerSimonian and Laird (1986) [12]. Otherwise, a fixed effect model was used as previously described by Mantel-Haenszel (1959) [13]. The restricted cubic splines method [14], as described by Orsini *et al.*, was performed to test the potential nonlinear relations using three fixed knots at 10%, 50% and 90% of potassium levels, respectively. Publication bias was assessed using a funnel plots and a weighted Egger’s test [15]. All analyses were performed using software STATA version 12.0 (StataCorp LP, College Station, TX, USA). This meta-analysis is registered at http://www.crd.york.ac.uk/PROSPERO/asCRD42015029266.

## 3. Results

### 3.1. Characteristics of the Identified Studies

**Figure 1:**
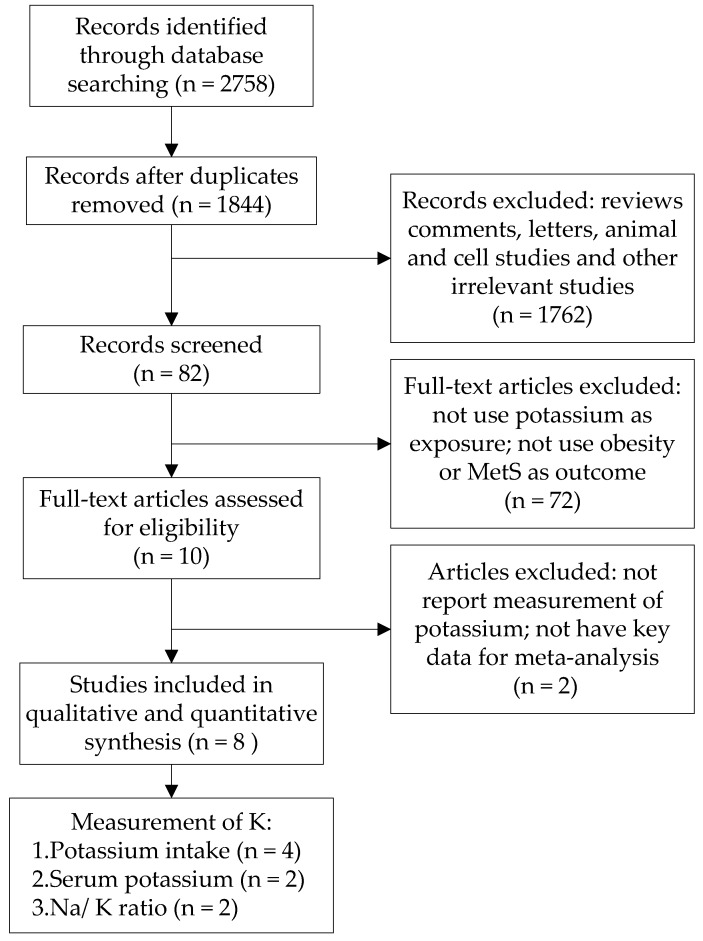
Flowchart of search strategy.

The initial search identified a total of 2758 potentially relevant studies. After removing duplicates and screening of titles and abstracts, we identified 82 studies for full-text assessment. Based on our inclusion criteria and exclusion criteria, 8 studies [10,16,17,18,19,20,21,22] (7 cross-sectional studies and 1 cohort study) were included in the systematic review and meta-analysis, as shown in Figure 1. There were 51,702 participants from four different countries (3 studies from China, 2 studies from Japan, 2 studies from Korea and 1 study from USA), consisting of 15,527 obesity cases and 10,482 MetS cases. Six studies recruited both male and female participants, and the other two studies recruited only females. Four studies used potassium intake as exposure, two studies used serum potassium as exposure, and the last two used urinary sodium-to-potassium ratio as exposure. The four studies regarding potassium intake as exposure were included in meta-analysis. Detailed information of each study is summarised in Table 1.

### 3.2. Potassium and Obesity

**Figure 2:**
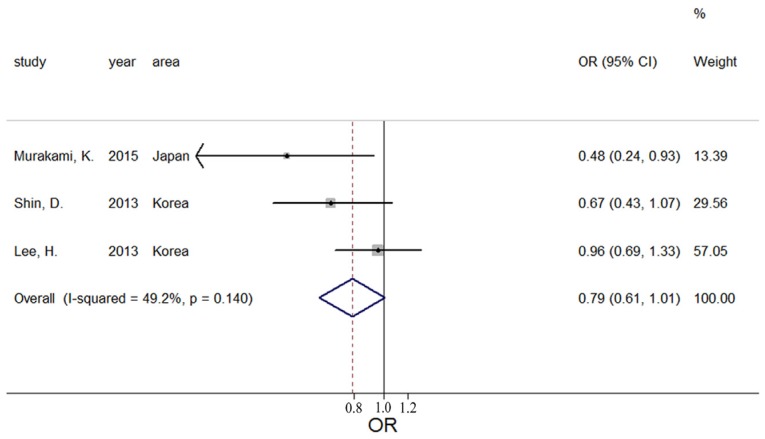
Forest plot of meta-analysis on potassium and obesity.

**Figure 3:**
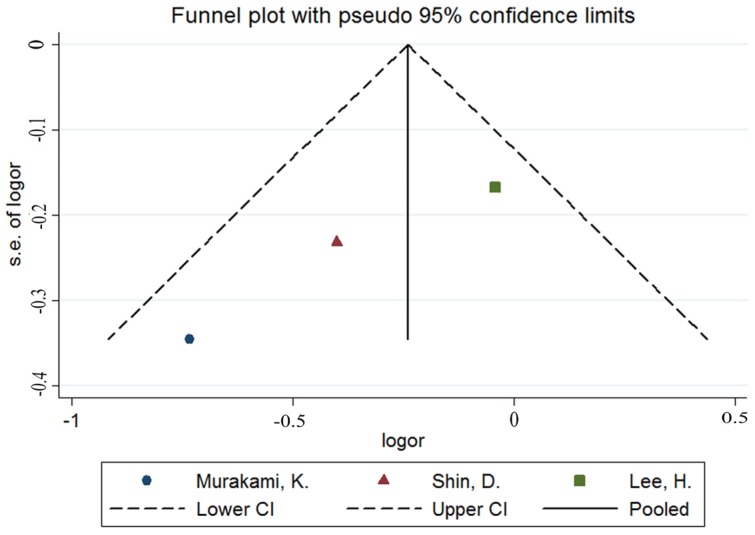
Funnel plot of meta-analysis on potassium and obesity.

**Figure 4:**
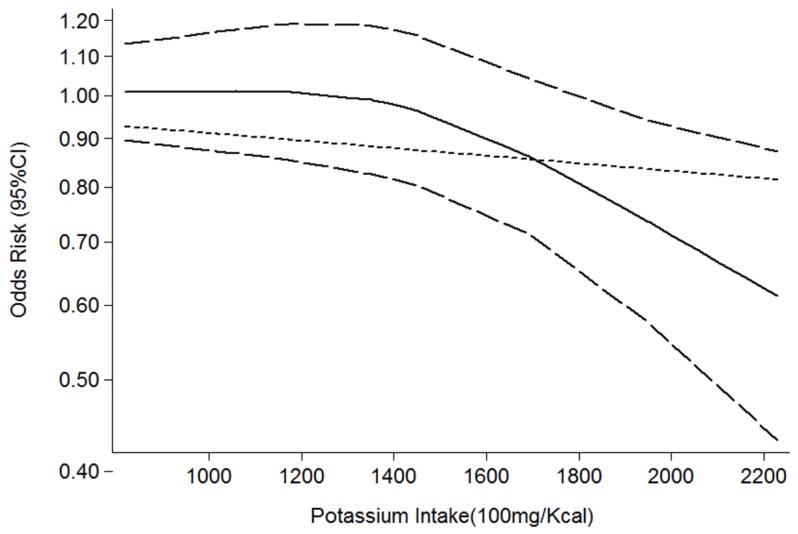
Summary nonlinear dose-response curves: potassium and obesity.

Overall, three estimates of the relationship between high potassium and obesity were included in the meta-analysis. The results of the pooled analysis indicated that high potassium intake was not associated with obesity (pooled OR = 0.78; 95% CI: 0.61–1.01) with homogeneity (Q = 3.94; *p* = 0.140; I^2^ = 49.2%, as shown in Figure 2). The funnel plot was symmetric (Figure 3) to exclude publication bias. In nonlinear analysis, three groups of data were included [10,16,19]. We found an obvious downward curve in the nonlinear graph (*p* = 0.025 for nonlinearity, Figure 4), indicating a protective effect of adequate potassium intake on obesity.

Two studies performed by Sun *et al.* [17,18] revealed that high serum could reduce the risk of obesity (OR = 0.76, 95% CI: 0.70–0.82, and OR = 0.63, 95% CI: 0.52–0.77, respectively). Additionally, one study performed by Jain *et al.* [21] in USA showed that the patients with the highest potassium intake relative to sodium had the lowest risk of obesity, and this effect was most pronounced for African American subjects. Ge *et al.* [22] study revealed that urinary sodium-to-potassium ratio was also associated with obesity independently, and high sodium-to-potassium ratio could increase the risk of obesity, which was similar to the results of Jain *et al*.

### 3.3. Potassium and Metabolic Syndrome

**Figure 5:**
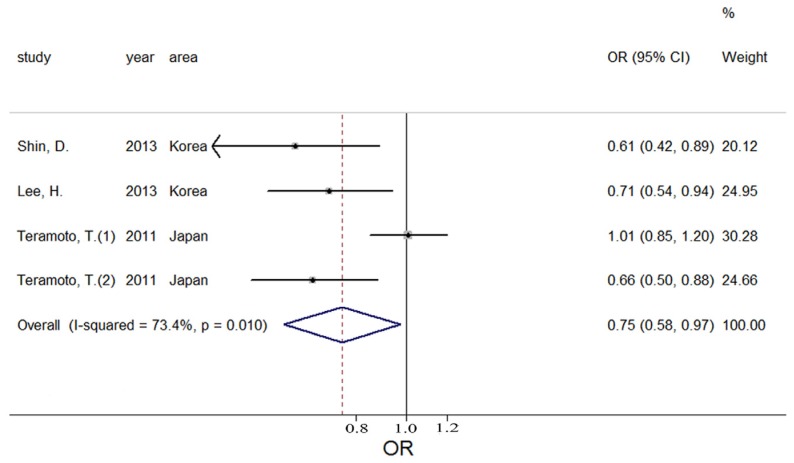
Forest plot of meta-analysis on potassium and MetS.

**Figure 6:**
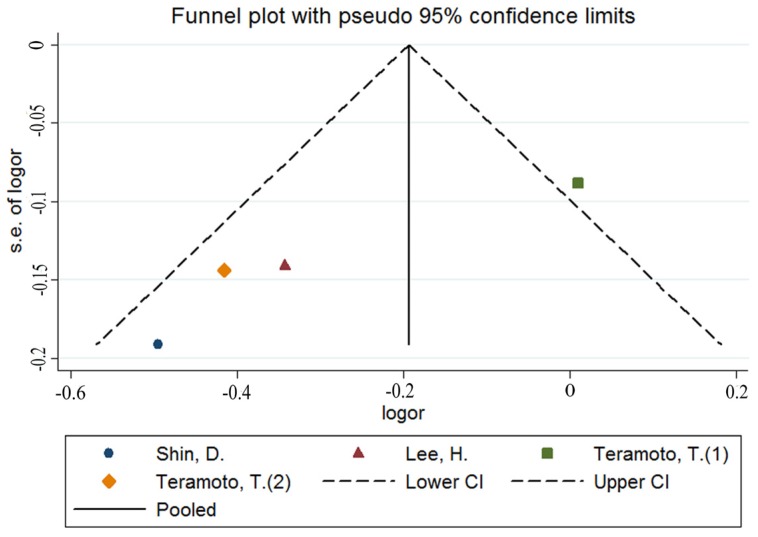
Funnel plot of the meta-analysis on potassium and MetS.

**Figure 7:**
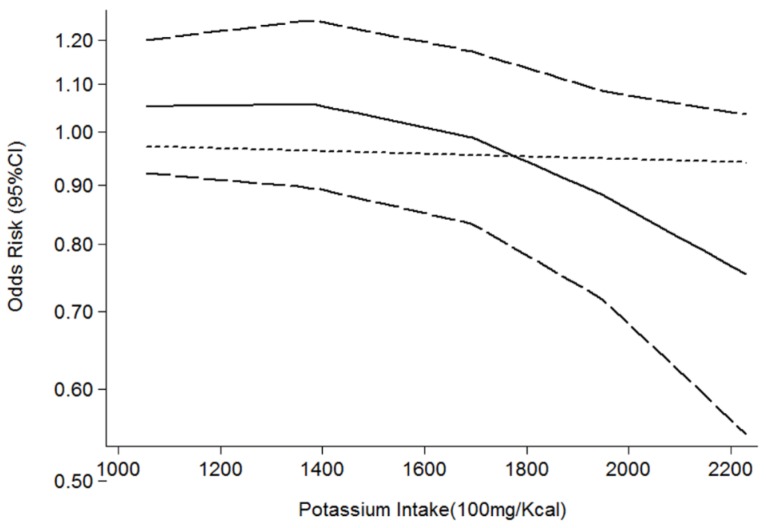
Summary nonlinear dose-response curves: potassium and MetS.

A total of four estimates were included in the meta-analysis. All the included studies had the same define of MetS such as the presence of central obesity plus dyslipidemia (triglyceride level ≥ 150 mg/dL; HDL cholesterol level < 40 mg/dL) and/or blood pressure ≥ 130/85 mmHg or fasting glucose level ≥ 100 mg/dL. We incorporated the four estimates into the meta-analysis. The pooled OR was 0.75 (95% CI: 0.50–0.97) with moderate heterogeneity (Q = 11.29; *p* = 0.010; I^2^ = 73.4%, as shown in Figure 5), indicating that potassium intake was associated with MetS at the highest compared with the lowest category. The heterogeneity could be visualized using an asymmetric funnel plot (Figure 6). In nonlinear analysis, two groups of data were included [10,19]. We also found an obvious downward curve in the nonlinear graph (*p* = 0.056 for nonlinearity, Figure 7), indicating a protective effect of adequate potassium intake on MetS.

A study performed by Sun *et al.* [17] also indicated that serum potassium was associated with MetS. However, no research paper was found about the relationship between urinary sodium-to-potassium ratio and MetS.

## 4. Discussion

The results of meta-analysis and systematic review indicated potassium intake should not be investigated simply, and sodium-to-potassium ratio seemed to be a more sensitive index to obesity for the joint consideration of both sodium and potassium. In addition, the participants in the highest category of potassium intake had lower odds for metabolic syndrome compared to those in the lowest category (pooled OR = 0.75; 95% CI: 0.50–0.97). Consistent with these findings, our nonlinear analysis revealed a protective effect of adequate potassium intake on obesity and MetS. Additionally, high potassium intake may play a more important role in females than males.

Recently, the relationship between potassium intake and obesity/MetS was controversial. A study performed by Murakami *et al.* [16] indicated that higher potassium intake is associated with a lower risk of obesity, while studies performed by Shin *et al.* [10] and Lee *et al.* [19] found that potassium intake had a null effect on obesity. According to metabolic syndrome, Shin *et al.* [10] showed that high potassium intake was a protective factor, and this finding was consistent for both sexes. Nevertheless, Teramoto *et al.* [20] revealed that potassium was more correlated with MetS in females than in males. The urinary sodium-to-potassium ratio was found to be associated with obesity robustly, which also influenced blood pressure [23,24]. However, no articles reported the association between sodium-to-potassium ratio and MetS.

Our meta-analysis included 51,702 subjects from four different countries ranging from 2011 to 2015 and consisted of 15,527 obesity and 10,482 MetS cases. Nevertheless, most studies were performed in Asia (China, Japan and Korea) were in Asia. There are no relevant studies in the European countries and other areas that have high rates of obesity and metabolic syndrome. However, all of the included studies in our meta-analysis were published within the past five years. This finding indicates that the association between potassium and obesity/MetS appears to be a relatively new topic for nutrients and public health. However, these recent studies were confined to Asia and USA, and have small-scale limitations. We propose that it is necessary to perform global research studies using a larger number of participants in the future with prospective design or random controlled trials. Sodium-to-potassium ratio is probably also a sensitive index to evaluate the risk of obesity and MetS in addition to potassium intake, although more studies are needed to verify the results.

In this study, four studies employed dietary surveys to assess potassium intake, while two studies used serum potassium as exposure and the last two used urinary sodium-to-potassium ratio as exposure. We could not conduct meta-analysis for serum potassium and sodium-to-potassium ratio for the lack of sufficient relevant studies. Hence, a systematic review was performed to describe the relationship between serum potassium/sodium-to-potassium ratio and obesity/MetS. Murakami *et al.* [16] showed that high potassium assessed from 24 h urinary excretion was also associated with obesity. Nevertheless, the results of Ge *et al.* [25] did not indicate a significant association between 24 h urinary potassium excretion and MetS. Due to the lack of sufficient relevant studies, we did not perform the meta-analysis between 24 h urinary potassium excretion and obesity/MetS.

The precise mechanism between potassium intake and obesity/MetS is unclear. Central obesity is a component of metabolic syndrome, and the mechanisms of obesity and MetS are homogeneous. Obesity has been shown to be associated with potassium channel function [26,27]. Potassium can affect carbohydrate accumulation and glucose homeostasis [28,29] and plays a critical role in insulin secretion and carbohydrate metabolism [28,30]. Moreover, it is also a predictor of incident diabetes [31]. Dietary potassium intake has also been inversely correlated with blood pressure [24,32,33]. However, the protective effect of high potassium intake on obesity may be due to the high intake of fruits and vegetables, which are major sources of potassium [34] and have also been shown to be beneficial to metabolic syndrome [35].

This study has several limitations. First, a small number of studies were included in the study. Only four relevant studies and seven estimates were incorporated into the pooled analysis, and all of the relevant articles included in the meta-analysis are cross-sectional studies, which are not as scientific as prospective observational studies and random controlled trials. Second, an insufficient number of relevant estimates weaken the accuracy of the results of nonlinear dose-response analysis, and we have limited power to perform a subgroup analysis of some potential confounding factors. Third, 24 h dietary recall may not be accurate to assess the precise dietary intake due to errors in estimating the nutriment size and faults in memory. In addition, the relevant studies did not apply the same food items in the frequency questionnaire to survey the dietary potassium intake. Although recent studies revealed the inverse relationship between serum potassium and obesity/MetS, this condition may be contributed by the subclinical elevations of cortisol, and be the results of hypercortisolism or hyperaldosteronism rather than potassium intake. Prospective studies with a large sample size and accurate assessment of potassium intake are needed to confirm or update our results. Additionally, the effects of sodium-to-potassium ratio on obesity and MetS are recommended to be researched. We will update the meta-analysis in the future when high quality studies (prospective cohort studies and RCTs) are reported.

Our study also has several strengths. To the best of our knowledge, this is the first systematic review and meta-analysis to investigate the overall effect of potassium on the risk of obesity and MetS, and our study reviewed the effect of different measurement of potassium (intake, serum and sodium-to-potassium ratio) on obesity and MetS in detail. Furthermore, we performed nonlinear does-response analysis using the restricted cubic splines method to test the potential nonlinear relationships. These results suggest a daily intake of potassium that is larger than 2200 mg/Kcal to reduce the risk of obesity and MetS. Moreover, we applied strict inclusion criteria to reduce selection bias and improve the reliability of our conclusions.

## 5. Conclusions

Potassium is correlated with central obesity and metabolic syndrome. Adequate daily potassium intake can reduce the risk of obesity and MetS. In addition, these findings support adequate intake of fruits and vegetables, which are major sources of potassium. Furthermore, additional relevant studies are needed to examine the underlying mechanism.

## Figures and Tables

**Table 1:** Characteristics of studies included in the meta-analysis.

Study	Country	Age	Participants	Cases	Measurement of K	Gender	Outcome	OR (95% CI)	Controlled Factor
Murakami, K. (2015)	Japan	18–22 y	1043	136	Intake	Women	Obesity	0.48 (0.24, 0.93)	year, region, municipality level, residential status, living alone or living with others, alcohol drinking, smoking, physical activity, other nutrients
Shin, D. (2013)	Korea	≥20 y	7542	1568	Intake	M and F	Obesity	0.67 (0.43, 1.07)	total energy, carbohydrate, total fat, fibre, vitamin C, and sodium intakes
				1269	Intake	M and F	MetS	0.61 (0.42, 0.89)	
Lee, H. (2013)	Korea	Mean 45.3 y	9911	3885	Intake	Women	Obesity	0.96 (0.69, 1.33)	age, BMI, alcohol intake, exercise, education, income, residential area, frequency of fruit intake, energy, and carbohydrate energy ratio
				2012	Intake	Women	MetS	0.71 (0.54, 0.94)	
Teramoto, T. (2011)	Japan	Mean 64.9 y	4656	2277	Intake	Men	MetS	1.01 (0.85, 1.20)	Unadjusted
			4929	943	Intake	Women	MetS	0.66 (0.50, 0.88)	
Ge, Z. (2015)	China	18–69 y	1906	992	Na/K ratio	M and F	Obesity	0.74 (0.56, 0.98)	age, sex, education, urbanization, leisure-time physical activity, alcohol consumption, smoking, hypertension, antihypertensive treatment, fasting plasma glucose and TAG
Jain, N. (2014)	USA	Mean 44 y	2782	890	Na/K ratio	M and F	Obesity	0.43 (0.15, 0.72)	age, sex, race, DM, SBP, DBP, and serum glucose and triglyceride concentrations
Sun, K. (a) (2014)	China	≥40 y	10,341	4361	Serum	M and F	Obesity	0.76 (0.70, 0.82)	age, sex, BMI, current smoking and drinking status; other components of metabolic syndrome as dichotomised variables
				3981	Serum	M and F	MetS	0.68 (0.53, 0.86)	
Sun, K. (b) (2014)	China	Mean 58.6 y	8592	3695	Serum	M and F	Obesity	0.63 (0.52, 0.77)	age, sex, BMI, current smoking status, use of drug, FPG, HbA1C, TG, TC, LDL-C, HDL-C, SBP, DBP, ALT, AST, serum sodium, serum magnesium, eGFR and HOMA-IR

Notes: BMI: body mass index; FPG: fasting plasma glucose; HbA1C: haemoglobin A1c; TG: triglycerides; TC: total cholesterol; LDL-C: low-density; HDL-C: high-density lipoprotein; SBP: systolic blood pressure; DBP: diastolic blood pressure; eGFR: estimated glomerular filtration rate; HOMA-IR: homoeostasis model assessment of insulin resistance. M and F: males and females; Y: years; DM: diabetes mellitus.
